# Prediction of skin color, tanning and freckling from DNA in Polish population: linear regression, random forest and neural network approaches

**DOI:** 10.1007/s00439-019-02012-w

**Published:** 2019-04-12

**Authors:** Katarzyna Zaorska, Piotr Zawierucha, Michał Nowicki

**Affiliations:** 1 Department of Histology and Embryology, University of Medical Sciences, 60-781 Poznan, Poland; 2 Department of Anatomy, University of Medical Sciences, 60-781 Poznan, Poland

## Abstract

Predicting phenotypes from DNA has recently become extensively studied field in forensic research and is referred to as Forensic DNA Phenotyping. Systems based on single nucleotide polymorphisms for accurate prediction of iris, hair and skin color in global population, independent of bio-geographical ancestry, have recently been introduced. Here, we analyzed 14 SNPs for distinct skin pigmentation traits in a homogeneous cohort of 222 Polish subjects. We compared three different algorithms: General Linear Model based on logistic regression, Random Forest and Neural Network in 18 developed prediction models. We demonstrate Random Forest to be the most accurate algorithm for 3- and 4-category estimations (total of 58.3% correct calls for skin color prediction, 47.2% for tanning prediction, 50% for freckling prediction). Binomial Logistic Regression was the best approach in 2-category estimations (total of 69.4% correct calls, AUC = 0.673 for tanning prediction; total of 52.8% correct calls, AUC = 0.537 for freckling prediction). Our study confirms the association of rs12913832 (*HERC2*) with all three skin pigmentation traits, but also variants associated solely with certain pigmentation traits, namely rs6058017 and rs4911414 (*ASIP*) with skin sensitivity to sun and tanning abilities, rs12203592 (*IRF4*) with freckling and rs4778241 and rs4778138 (*OCA2*) with skin color and tanning. Finally, we assessed significant differences in allele frequencies in comparison with CEU data and our study provides a starting point for the development of prediction models for homogeneous populations with less internal differentiation than in the global predictive testing.

**Electronic supplementary material:**

The online version of this article (10.1007/s00439-019-02012-w) contains supplementary material, which is available to authorized users.

## Introduction

DNA phenotyping is recently one of the most relevant study areas in the forensic field. Predictions of human externally visible characteristics (EVCs) are possible through genotyping of single nucleotide polymorphisms (SNPs). Most of the EVCs, e.g., human pigmentation or hair and facial morphology, are complex polygenic and multifactorial traits, yet they are highly heritable and can be classified into easily described categories (Pulker et al. 2007; Kastelic and Drobnič 2012; Walsh et al. 2013; Liu et al. 2012). Though EVC prediction of a human phenotype’s characteristics from DNA markers requires a probabilistic method, it provides an important and very useful tool in both criminal network as well as in archeological anthropology studies and it refers to as “DNA intelligence” (Kayser and Schneider 2009; Dario et al. 2015). There is an increasing knowledge on genetic factors that explain differences in human morphological traits and SNPs are considered to affect human phenotypic variation the most (Kayser and de Kniff 2011; Wei et al. 2014).

Pigmentation is one of the most differentiated human phenotypic traits, especially among Europeans (Bouakaze et al. 2009; Walsh et al. 2012). So far, the best informative pigmentation SNPs have been described for the iris color (six SNPs) and hair color (24 SNPs). These SNP markers display prediction accuracy of over 90% for blue and brown eye color as well as 70–87.5% for hair color and are termed as IrisPlex (Walsh et al. 2013) and HIrisPlex (Walsh et al. 2011). But, still, little is known about the other EVC traits. Skin color is considered as an adaptive trait and melanin synthesis is a complex process, since multiple genes as well as other factors, such as age, diseases, drugs and environmental factors can contribute to the final outcome (Spichenok et al. 2011; Srettabunjong et al. 2016). Several candidate gene loci have been identified to be presumably associated with skin pigmentation traits in people of European ancestry as well as to differ among geographical populations. Only recently, a profound and much broader discussion on the genetic background of human pigmentation diversity has emerged (Quillen et al. 2019).

Based on a special emphasis that has been given to distinct variants with presumable greatest relevance for skin pigmentation traits in Europeans, especially those of Central/Eastern Europe, on previous studies, here, we have chosen 14 SNPs in nine genes for testing the association with skin color, skin susceptibility to sunburns and freckling features in the homogeneous Polish population in a total of 18 primary prediction models based on three distinct mathematical approaches.

## Materials and methods

### Sample collection and DNA extraction

A total of 222 (90 males and 132 females) unrelated individuals from Poland, aged 20–63 (mean 26, *σ* = 9.8) were recruited for our study in 2016. Oral swabs [FloqSwabs hDNA Free (COPAN)] were collected and genomic DNA was extracted using ExtractMe DNA Swab & Semen Kit (Blirt S.A.) according to the manufacturer’s instruction.

### Study design

All individuals gave informed consent prior to sample donation. They were asked to fill in the questionnaire that included the basic information, such as gender, age and ethnic origin, as well as particular phenotypic features (individuals aged > 30 years were asked about the phenotypic features at their mid-20s) (Srettabunjong et al. 2016), such as the iris and hair color, skin color and tone, susceptibility to sunburns and the presence of freckles (described as solar lentigines and ephelides). These traits were graded into the following categories: for iris color: brown/blue (or gray)/intermediate (including green), for hair color: black/brown/red/blonde, for skin color: dark (olive)/medium/light (pale), for tanning/skin sensitivity to sun: high susceptibility to sunburns/initial sunburns (but turning brown)/moderate tanning (without sunburns)/quick tanning, for freckling: severe freckling/moderate freckling/non-freckled skin. Severe freckling referred to an abundant freckling present on the face and arms/shoulders (also accompanied by the presence of freckles on other body areas with limited or no exposure to sun during any season), while moderate freckling referred to a mild freckling pattern found on the face and arms but not on other areas of the body. Skin color referred to the inner part of upper arm, according to the regime recommended by Stokowski et al. (2007). Since we evaluated skin pigmentation traits in this study, the iris and hair color was additional information and was not included in the prediction modeling. The entire experimental group was divided into two subgroups named training and testing. Training group consisted of 150 individuals (75 males, 75 females) randomly selected from 222 individuals enrolled in this study, whereas testing group comprised the remaining 72 individuals (15 males, 57 females).

### SNP selection and genotyping

14 autosomal SNPs affecting the general pigmentation were chosen for genotyping in our study. They were: (a) rs12913832 in *hect domain and RCC1*-*like domain 2* (*HERC2*) gene, (b) rs1800407, (c) rs7495174, (d) rs4778241 and (e) rs4778138 in the *oculocutaneous albinism II* (*OCA2*) gene, (f) rs12896399 in *solute carrier family 24*, *member 4* (*SLC24A4*) gene, (g) rs16891982 in *solute carrier family 45*, *member 2* (*SLC45A2*) gene, (h) rs12203592 in *interferon regulatory factor 4* (*IRF4*) gene, (i) rs1393350 in *tyrosinase* (*TYR*) gene, (j) rs731236 in *vitamin D receptor* (*VDR*) gene, (k) rs6058017, (l) rs1015362 and (m) rs4911414 in *Agouti signaling protein* (*ASIP*) gene, (n) rs1805007 in *melanocortin 1 receptor* (*MC1R*) gene. We chose the SNPs based on their documented association with pigmentation traits within Europe and not the ones that have been reported as an Ancestry Informative Markers (AIM) that correlated more with an ethnic descent rather than with a visible trait, e.g., rs1426654 in *SLC24A5* (Dario et al. 2015; Bouakaze et al. 2009; Lao et al. 2007). All marker details including primer sequences and concentrations can be found in Supplementary Table 1. Of those 14 SNPs, 6 make up the IrisPlex (a, b, f, g, h, i) and 1 is included in the HIrisPlex (*n*) and they were genotyped according to Walsh et al. (2011, 2013, respectively). The remaining seven SNPs were genotyped in a single multiplex two step PCR. The free web-based software BatchPrimer3 v1.0 was used to design PCR and single base extension (sbe) reaction primers using parameters according to others (Kaderali et al. 2003; van Oven et al. 2011). To ensure the minimal interaction between the primers in the multiplex, they were checked in OligoAnalyzer v3.1 using parameters according to Vallone and Butler (2004). The protocol encompassed a single multiplex PCR in a 10-μl reaction mixture containing 1 ng genomic DNA, 1U FastStart Taq Polymerase (Roche), 1xPCR buffer with 1.5 mM MgCl_2_, 1 × GC-rich buffer, 200 μM of each dNTP and adequate concentration of forward and reverse primers. Thermocycling conditions were: 95 °C for 10 min for 1 cycle, and 95 °C for 30 s, 59 °C for 30 s, 72 °C for 30 s for 33 cycles, followed by 72 °C for 15 min. The PCR product was cleaned using ExoI/rSAP and SmartCut buffer (New England Biolabs). This was followed by the multiplex sbe reaction using 1 μl cleaned product, 1 μl SNaPshot reaction mix (Applied Biosystems) and a desired concentration of sbe primers in a total volume of 5 μl. Thermocycling conditions were: 96 °C for 2 min, 25 cycles of 96 °C for 10 s, 50 °C for 5 s, 60 °C for 30 s. The SNaPshot reaction product was cleaned using rSAP and SmartCut buffer (New England Biolabs). Finally, all products were run on an ABI 3130 Genetic Analyzer (Applied Biosystems) with POP-7 on a 36 cm capillary length array and run parameters were optimized to increase sensitivity, i.e. the injection voltage of 2.5 kV for 10 s and run time of 600 s at 60 °C. GeneMapper v4.0 (Applied Biosystems) was used for allele calling.

### Statistical analysis

#### Population analysis

Haploview v4.2 was used to assess the linkage disequilibrium (LD) values for tested SNPs as well as to estimate whether the distribution of genotypes in the training group was consistent with Hardy–Weinberg equilibrium (HWE). The frequencies of alleles and genotypes for subjects, for phenotypes and for comparison with 1000 Genomes data of European Americans (CEU, Utah residents with Northern and Western European ancestry) (Genomes Project Consortium 2015) were assessed using the Fisher’s exact test with 95% CI. The calculations were performed both for 2-category level (binomial estimation) as well as 3- and 4-category levels (multinomial estimation). For the purpose of binomial estimation, the phenotype categories were adjusted as follows: for skin color: dark vs. non-dark (comprising moderate and light/pale), for tanning: sunburns (comprising high susceptibility and initial sunburns) vs. non-sunburns (comprising moderate and quick tanning) and for freckling: freckled skin (comprising severe and moderate freckling) vs. non-freckled skin. Correlation of three pigmentation traits was performed using Cramér’s *V* test where the result varied from 0 (corresponding to no association between the variables) to 1 (corresponding to complete association). All analyses were made using the R language v3.5.0 and RStudio IDE v1.1.383.

#### Prediction modeling

**Fig. 1:**
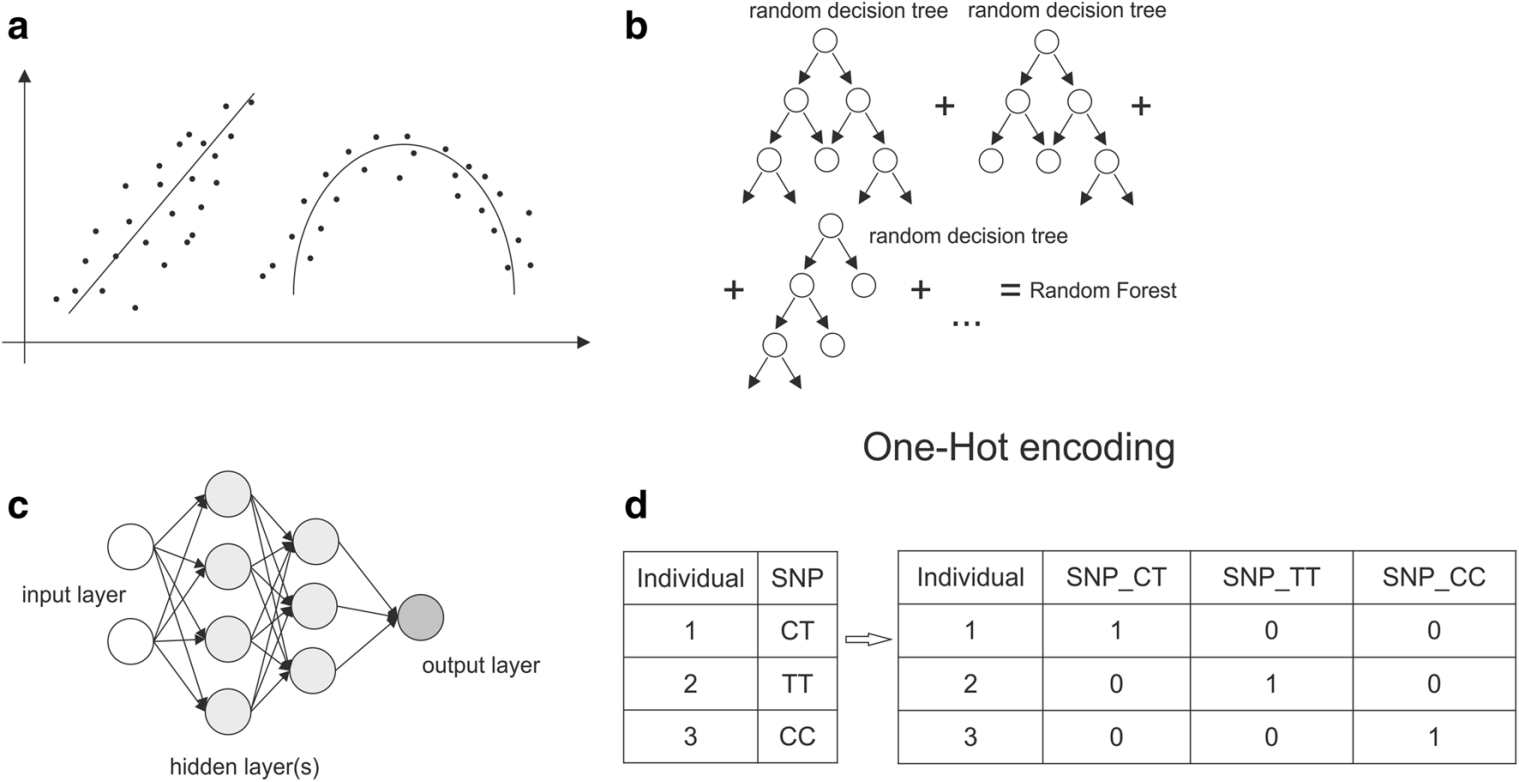
Machine learning algorithms used in this study. **a** General linear model, **b** random forest, **c** neural network, **d** one-hot encoding; descriptions in “Materials and methods” section in the text

Prediction modeling was performed on 150 individuals of the training group using machine learning (ML) approach. We compared three different algorithms: general linear model (GLM), random forest (RF) and neural network (NN). To avoid false-positive results and over-fitting of the model, all ML algorithms, analogically to the study group, used two data sets, namely training (to train the developed model) and testing (to evaluate how well the model recognized previously unknown data). Each algorithm uses a different approach, which is vividly shown in Fig. 1. Briefly, GLM (Fig. 1a) is based on mathematical estimation of curve that fits best to the data. For our categorical type of data, we used a subfamily of GLM called binomial logistic regression (BLR) for 2-category estimation and multinomial logistic regression (MLR) for 3- and more-category estimation. The final prediction was characterized by the sensitivity, specificity, total number of correct calls, LogLoss and AUC values, and the importance of single predictor’s contribution to the model was described as coefficient β in function equation describing best-fitted curve. Next, RF uses a tree-like graph and gathers information from a given number of decision trees (Fig. 1b). Each tree further splits random data to get information about its structure to choose the best-fitting model. For categorical data, we used classification model and mode-type of results. The advantage of RF is that it does not over-fit the model. At last, NN mimics the function of brain neurons. Neural networks are represented as directed graphs, where each node (neuron) has a given number of input and output edges (Fig. 1c). Each edge is associated with weight, i.e., the number that can be tuned during an algorithm process. After each sample flow, which is processed by the H_2_O library, the weights are corrected to minimize the error rate. The optimization (starting point and self-learning) is performed in hidden layers of NN. For categorical variables, the model requires one neuron per each category level. Both RF and NN were defined by the sensitivity, total number of correct calls and LogLoss values, with percentage number of single SNP importance in each model. In addition, H_2_O package translated our input categorical data using one-hot encoding method (Fig. 1d). To find the best setup, each algorithm was tested in terms of several parameters called hyperparameters. The process of assessing ones is called grid search and is performed prior to prediction modeling. As grid search tasks are time-consuming, they were conducted on the supercomputer at PSNC (Poznan Supercomputing and Networking Center). All analyses were made using the R language v3.5.0 and RStudio IDE v1.1.383, with the following packages: H_2_O v3.20.0.8 (implementation of AI (Artificial Intelligence) methods), Dplyr v0.78 (implementation of the method used in data manipulation) and Readr v1.3.1 (support for the import of *.xls/xlsx files to R session).

## Results

### Phenotype and genotype characteristics

The frequencies of phenotypic traits are shown in Table 1. When compared with 1000 Genomes data of European Americans, there were significant differences in allele distribution for six SNP markers: rs4778241 (*p* = 0.0498), rs4778138 (*p* = 0.0337), rs731236 (*p* = 0.0097), rs12203592 (*p* = 0.0056), rs12896399 (*p* = 0.0010), rs1805007 (*p* = 0.0018) (data not shown). One SNP, rs7495174, turned out to have a heterozygote status in all training individuals and for that reason, it was excluded from further analyses. When genotype and allele frequencies were considered, we found significant difference between males and females for two *OCA2* variants, rs4778241 and rs4778138. The OR values and corresponding *p* values for 13 SNPs calculated for males and females in the training group can be found in Supplementary Table 2. When we analyzed the association of allele and genotype frequencies with the phenotype, we found significant results for 8 SNPs (rs12913832, rs4778241, rs4778138, rs16891982, rs12203592, rs6058017, rs4911414, rs1805007) for both binomial and multinomial estimations. All OR and corresponding p values with allele and genotype distribution in distinct phenotypes of skin pigmentation traits analyzed in this study can be found in Supplementary Table 3.

**Table 1:** Distribution of skin pigmentation phenotypes among study participants in the training group

Phenotype	No. (%) of individuals with phenotype			*p* value
	Males	Females	All	
Skin color				
Dark/olive	13 (17.3)	10 (13.3)	23 (15.3)	0.4977
Medium	38 (50.7)	33 (44)	71 (47.3)	0.4139
Light/pale	24 (32)	32 (42.7)	56 (37.4)	0.178
Dark	13 (17.3)	10 (13.3)	23 (15.3)	
Non-dark	62 (82.7)	65 (86.7)	127 (84.7)	0.4977
Total	75 (100)	75 (100)	150 (100)	
Tanning				
High susceptibility to sunburns	6 (8)	14 (18.7)	20 (13.3)	0.0613
Initial sunburns (but turning brown)	33 (44)	30 (40)	63 (42)	0.6198
Moderate	23 (30.7)	24 (32)	47 (31.4)	0.8603
Quick tanning	13 (17.3)	7 (9.3)	20 (13.3)	0.1553
Sunburns	39 (52)	44 (58.7)	83 (55.3)	
Non-sunburns	36 (48)	31 (41.3)	67 (44.7)	0.4119
Total	75 (100)	75 (100)	150 (100)	
Freckling				
Severe freckling	2 (2.7)	11 (14.7)	13 (8.8)	0.0197*
Moderate freckling	22 (29.3)	27 (36)	49 (32.6)	0.3847
Non-freckled skin	51 (68)	37 (49.3)	88 (58.6)	0.0212*
Freckled skin	24 (32)	38 (50.7)	62 (41.3)	
Non-freckled skin	51 (68)	37 (49.3)	88 (58.6)	0.0212*
Total	75 (100)	75 (100)	150 (100)	

Association was made using the Fisher’s exact test

Underline mimics a fraction bar; one should add up numbers above the bar to get a total number of individuals within a given category

Statistically significant * when *p* ≤ 0.05, ** when *p* ≤ 0.01, *** *p* ≤ 0.001

**Fig. 2:**
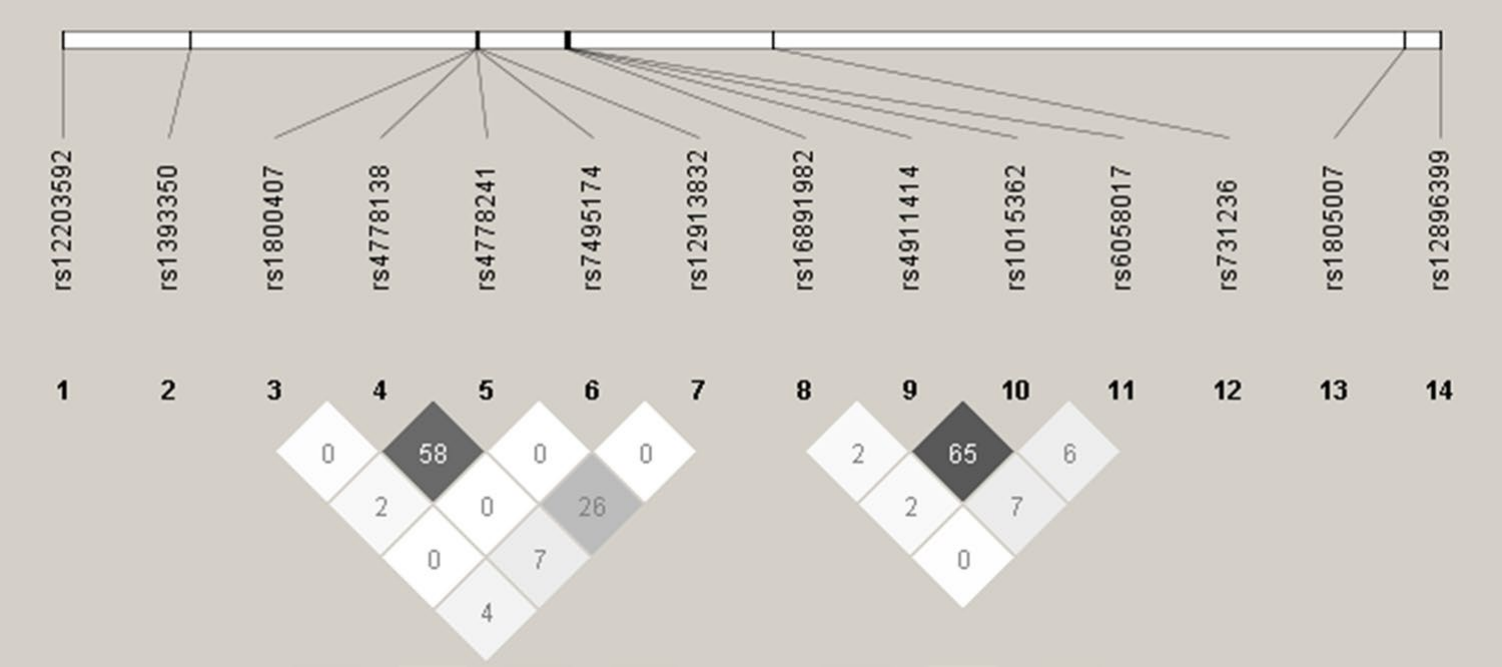
Heat plot showing pairwise measures of LD for all 14 SNPs tested for association with pigmentation trait in the training group. LD was considered significant when the value of the correlation coefficient *R*^2^ > 0.8

The pairwise measures of LD for 14 SNPs associated with pigmentation traits displayed by the heat plots using Gabriel et al. algorithm (Gabriel 2002) are shown in Fig. 2. LD was considered significant when the value of the correlation coefficient *R*^2^ > 0.8 (Barrett et al. 2005). The values of pairwise correlation between pigmentation traits are shown in Table 2. The highest value was observed for correlation between skin color and tanning (0.481); however, none of the results were significant.

**Table 2:** Cramér’s *V* test values of correlation between pairwise analyses for the three pigmentation traits tested in the study

	Skin color	Tanning	Freckling
Skin color	–	0.481	0.287
Tanning	0.481	–	0.247
Freckling	0.287	0.247	–

### Prediction modeling

**Fig. 3:**
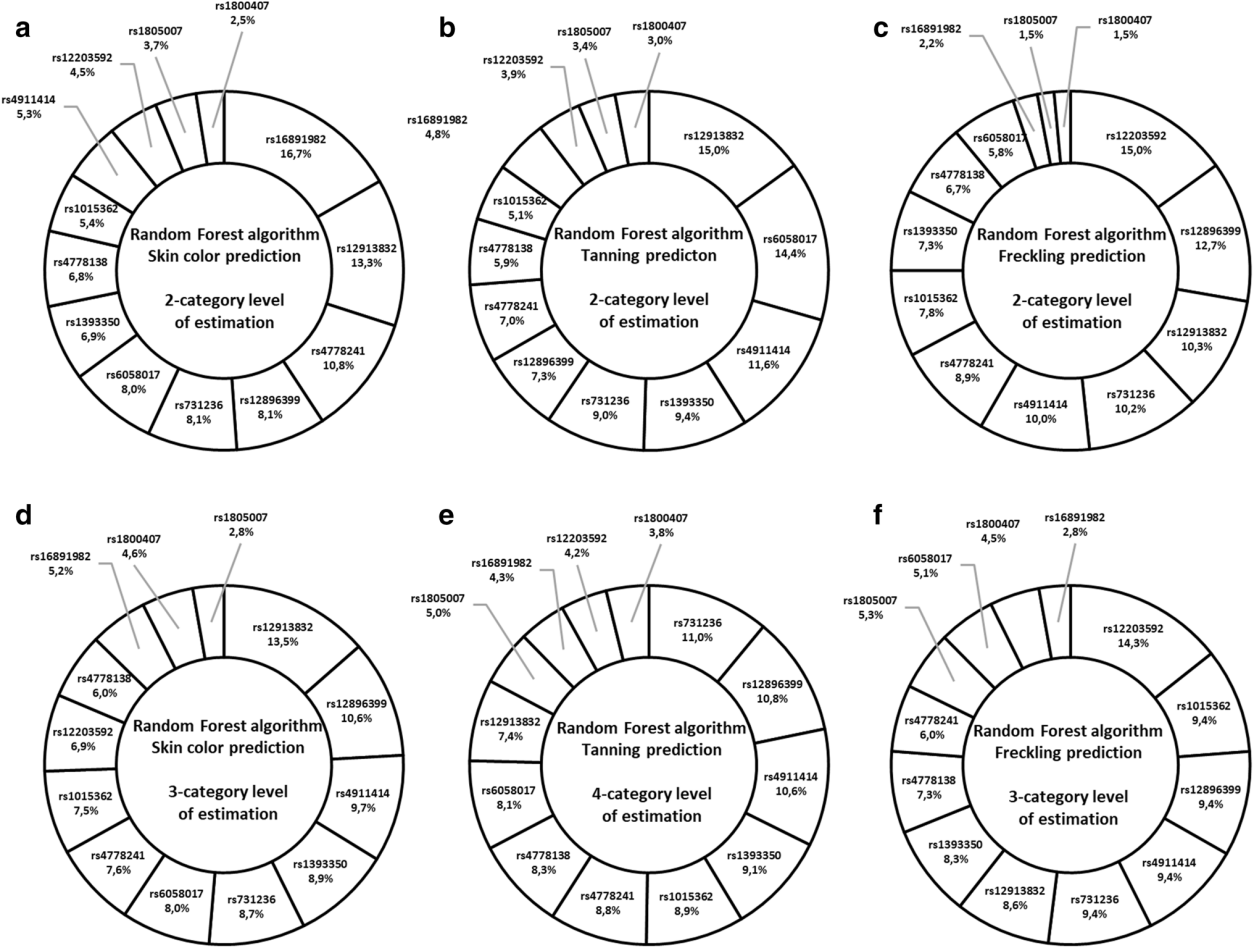
Representation of the percentage importance of SNPs genotyped in Random Forest prediction models. RF was performed on 13 SNPs tested for association with pigmentation traits for binomial (**a**–**c**) and multinomial (**d**–**f**) estimations. Rs7495174 is not present due to 100% heterozygous samples

**Fig. 4:**
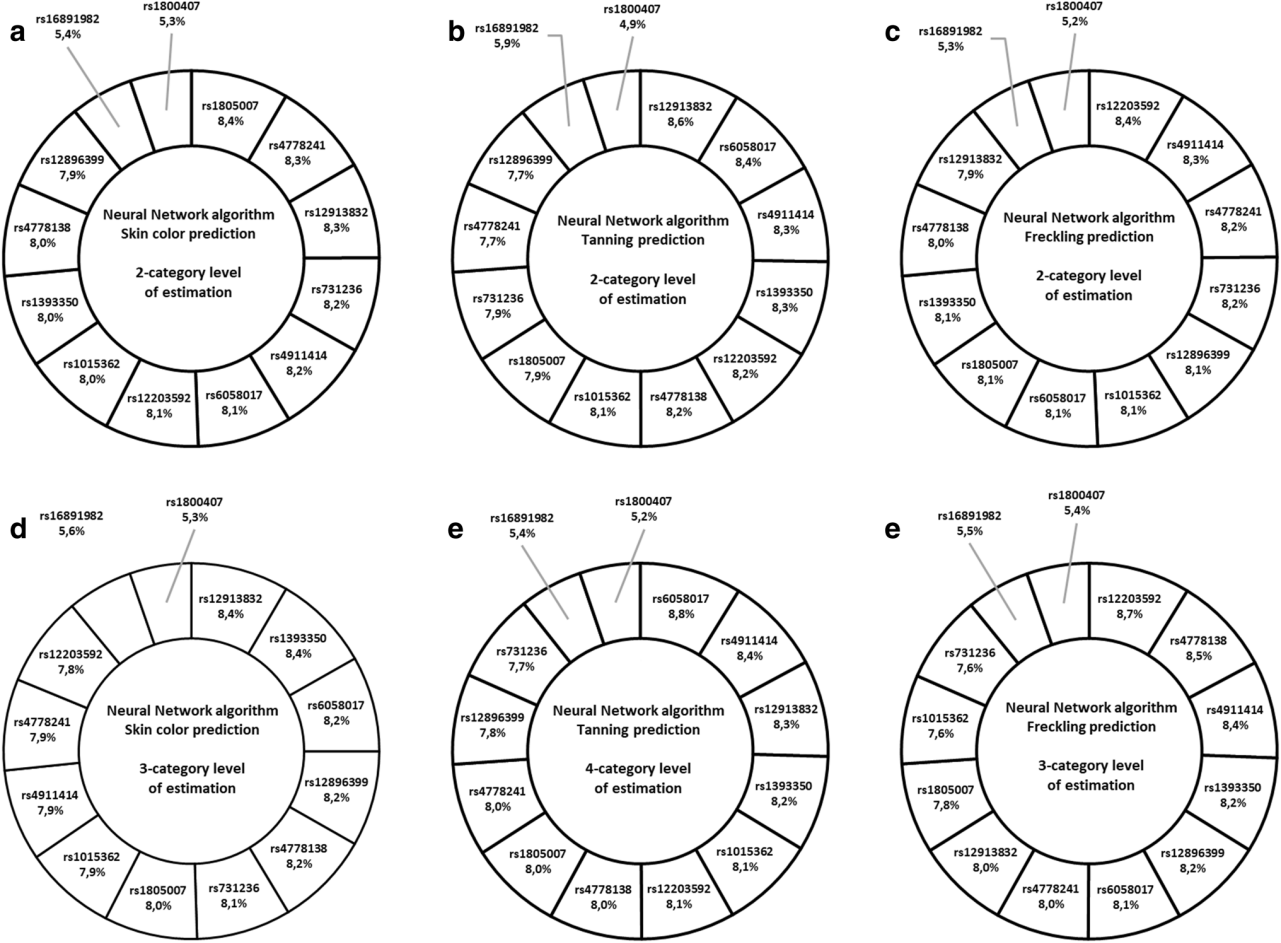
Representation of the percentage importance of SNPs genotyped in Neural Network prediction models. NN was performed on 13 SNPs tested for association with pigmentation traits for binomial (**a**–**c**) and multinomial (**d**–**f**) estimations. Rs7495174 is not present due to 100% heterozygous samples

In total, 18 prediction models were developed, namely 6 separate models for binomial and multinomial estimations for each algorithm tested. To specify the best-fitting model, we conducted a grid search over distinct hyperparameters that were specific to each algorithm. They were: for RF—number of trees, maximum depth of trees, minimal rows of features in each tree, sample rate and column sample rate per tree, and for NN—activation function, number of hidden layers as well as neurons in each layer, number of epochs (number of times algorithm must pass forward and backward on the entire data set), regularization level and model scoring interval. MLR was iterated over regularization of alpha parameter and that approach was supported by the a priori knowledge about the distribution family, i.e., multinomial for MLR, and the type of data, which in this case reduced the number of tested hyperparameters. The ultimate hyperparameters’ values that were applied to prediction modeling were different for each model and detailed summary of the results is shown in Supplementary Table 4. The parameters of prediction are shown in Table 3. All models were tested using tenfold cross validation. Additionally, we illustrated the importance of each SNP in developed prediction models in the form of pie charts. Figures 3 and 4 demonstrate the assembly of charts for RF and NN, respectively. The contribution of SNPs to BLR prediction of tested traits is demonstrated in Table 4. The coefficient *β* values for MLR prediction were negligibly small (LogLoss value < 0.0001) and did not contribute any [valuable] information gain from the model.

**Table 3:** Performance of the developed prediction models for three different algorithms (GLM, RF, NN) for 2-category estimation (binomial) and for 3- and 4-category estimations (multinomial) assessed for pigmentation traits

Parameters of prediction	Prediction model type					
	Binary logistic regression		Random forest		Neural network	
	Train^a^	Test^b^	Train	Test	Train	Test
Binomial prediction (2-category estimation level)						
Non-dark skin prediction sensitivity %	100% (127/127)	100% (69/69)	99.2% (126/127)	100% (69/69)	95.3% (121/127)	100% (69/69)
Non-dark skin prediction specificity %	39.1% (9/23)	0% (0/3)	78.3% (18/23)	0% (0/3)	52.2% (12/23)	0% (0/3)
Total number of correct calls %	90.7% (136/150)	96% (69/72)	96% (144/150)	96% (69/72)	88.7% (133/150)	96% (69/72)
AUC	0.921	0.611	0.984	0.519	0.903	0.587
LogLoss	0.226	0.842	0.229	0.255	0.324	0.457
Tanning prediction sensitivity %	85.5% (71/83)	83.3% (30/36)	94% (78/83)	80.5% (29/36)	83.1% (69/83)	88.9% (32/36)
Tanning prediction specificity %	71.6% (48/67)	55.6% (20/36)	74.6% (50/67)	47.2% (17/36)	59.7% (40/67)	33.3% (12/36)
Total number of correct calls %	79.3% (119/150)	**69.4% (50/72)**	86.7% (130/150)	63.9% (46/72)	72.7% (109/150)	61.1% (44/72)
AUC	0.846	0.673	0.914	0.621	0.791	0.593
LogLoss	0.467	0.757	0.509	0.682	0.637	0.894
Freckling prediction sensitivity %	74.2% (46/62)	100% (38/38)	83.9% (52/62)	100% (38/38)	64.5% (40/62)	97.4% (37/38)
Freckling prediction specificity %	81.8% (72/88)	0% (0/34)	96.6% (85/88)	0% (0/34)	81.8% (72/88)	17.6% (6/34)
Total number of correct calls %	78.7% (118/150)	**52.8% (38/72)**	91.3% (137/150)	52.8% (38/72)	74.7% (112/150)	60% (43/72)
AUC	0.818	0.537	0.956	0.575	0.773	0.565
LogLoss	0.51	1.023	0.416	0.741	1.184	1.95

Parameters of prediction	Prediction model type					
	Multinomial logistic regression		Random forest		Neural network	
	Train	Test	Train	Test	Train	Test
Multinomial prediction (3- and 4-category estimation level)						
Light/pale skin color prediction sensitivity %	0% (0/56)	0% (0/26)	71.43% (40/56)	30.8% (8/26)	37.5% (21/56)	15.4% (4/26)
Moderate skin color prediction sensitivity %	100% (71/71)	100% (43/43)	97.2% (69/71)	79.1% (34/43)	93% (66/71)	76.7% (33/43)
Dark/olive skin color prediction sensitivity %	0% (0/23)	0% (0/3)	60.87% (14/23)	0% (0/3)	34.8% (8/23)	33.3% (1/3)
Total number of correct calls %	47% (71/150)	60% (43/72)	82% (123/150)	**58.3% (42/72)**	63.3% (95/150)	52.8% (38/72)
LogLoss	1.009	0.881	0.646	0.868	0.846	0.937
High susceptibility to sunburn prediction sensitivity %	0% (0/20)	0% (0/5)	45% (9/20)	0% (0/5)	25% (5/20)	0% (0/5)
Initial sunburns prediction sensitivity %	100% (63/63)	100% (31/31)	98.4% (62/63)	83.9% (26/31)	41.3% (26/63)	29% (9/31)
Moderate tanning prediction sensitivity %	0% (0/47)	0% (0/27)	83% (39/47)	29.6% (8/27)	93.6% (44/47)	81.5% (22/27)
Quick tanning prediction sensitivity %	0% (0/20)	0% (0/9)	60% (12/20)	0% (0/9)	5% (1/20)	0% (0/9)
Total number of correct calls %	42% (63/150)	43% (31/72)	81.3% (122/150)	**47.2% (34/72)**	50.7% (76/150)	43% (31/72)
LogLoss	1.265	1.2	0.792	1.188	1.151	1.372
Severe freckling prediction sensitivity %	0% (0/13)	0% (0/8)	15.4% (2/13)	0% (0/8)	30.8% (4/13)	12.5% (1/8)
Moderate freckling prediction sensitivity %	0% (0/49)	0% (0/30)	59.2% (29/49)	13.3% (4/30)	77.6% (38/49)	50% (15/30)
Non-freckled skin prediction sensitivity %	100% (88/88)	100% (34/34)	98.9% (87/88)	94.1% (32/34)	61.4% (54/88)	53% (18/34)
Total number of correct calls %	59% (88/150)	47.2% (34/72)	83.3% (125/150)	**50% (36/72)**	64% (96/150)	47.2% (34/72)
LogLoss	0.89	0.99	0.542	1.025	0.771	1.126

Bold values indicate the model with the best predictive performance

^a^Train refers to the respective training group of subjects

^b^Test refers to the respective testing group of subjects

**Table 4:** Contribution of 13 SNPs selected for skin pigmentation prediction in terms of *β* coefficient and *p* value, within 2-category prediction models developed using BLR approach

SNP variant_genotype	Gene	Dark (*β*)	Dark (*p* value)	Non-sunburns (*β*)	Non-sunburns (*p* value)	Non-freckled skin (*β*)	Non-freckled skin (*p* value)
rs4778241_GT	*OCA2*	− 0.6038	0.5930	0.6745	0.3779	0.9779	0.1621
rs4778241_TT		24.9966	0.9519	1.7221	0.3945	− 11.7311	0.9528
rs4778138_CT	*OCA2*	− 12.4321	0.9575	− 1.0554	0.4993	1.9582	0.1902
rs4778138_TT		− 11.9705	0.9590	0.1724	0.9166	1.4411	0.3613
rs731236_AG	*VDR*	− 0.7766	0.3281	0.1879	0.6936	0.1867	0.6772
rs731236_GG		1.2076	0.4743	0.6686	0.3993	− 1.4646	0.0660
rs6058017_CT	*ASIP*	2.6165	0.9972	8.7797	0.9691	1.1557	0.5329
rs6058017_TT		2.5758	0.9972	10.5765	0.9628	2.0560	0.2712
rs1015362_CT	*ASIP*	− 1.3628	0.2179	− 1.2989	0.1067	− 0.6505	0.3471
rs1015362_TT		− 0.8790	0.5272	**− 4.6451**	**0.0047**	− 1.4580	0.1237
rs4911414_GT	*ASIP*	0.9741	0.3922	1.0922	0.1898	0.7492	0.2937
rs4911414_TT		1.2569	0.4521	**4.8503**	**0.0066**	**2.1952**	**0.0333**
rs12203592_CT	*IRF4*	− 0.5487	0.5990	1.0790	0.1090	**2.2296**	**0.0005**
rs12203592_TT		12.4492	0.9901	12.8093	0.9862	10.8671	0.9811
rs12913832_CT	*HERC2*	**− 2.2462**	**0.0109**	**− 1.0537**	**0.0462**	**− 1.1494**	**0.0267**
rs12913832_TT		− 15.2281	0.9479	− 2.1469	0.2054	− 0.5567	0.6399
rs1393350_CT	*TYR*	1.1068	0.2110	− 0.0639	0.8887	0.3682	0.4262
rs1393350_TT		12.7193	0.9749	20.6200	0.9427	1.5342	0.1786
rs12896399_GT	*SLC24A4*	1.3403	0.1319	0.1430	0.7939	0.4738	0.3344
rs12896399_TT		0.0962	0.9145	0.3720	0.5227	− 0.5365	0.3487
rs1805007_CT	*MC1R*	15.5136	0.9431	1.9599	0.0583	0.0449	0.9555
rs1805007_TT		12.4962	0.9900	11.5078	0.9876	10.7929	0.9812
rs16891982_GG	*SLC45A2*	**5.4121**	**0.0018**	**2.3170**	**0.0262**	0.9051	0.3123
rs1800407_GG	*OCA2*	− 13.6272	0.9534	− 0.4466	0.6049	− 0.0152	0.9860

*β* is calculated for the given phenotype. Bold values indicate statistically significant results

## Discussion

Three types of prediction models were applied and compared for best performance. The highest value of total correct calls for most predictions tested on both a 3- and 4-category levels was obtained with Random Forest and, slightly lower, with Neural Network, while GLM turned out to give the lowest predictive values. The latter produced 100% predictive values; however, it was due to the fact that all query phenotypes were classified as one phenotype in test prediction. On the other hand, GLM gave the best prediction values on a 2-category level, while RF and NN gave lower values, comparable with each other (Table 3).

For multinomial estimations, the prediction rates for skin color, tanning and freckling sensitivity were, respectively, 58.3%, 47.2% and 50% in RF, and were slightly higher than those obtained in NN. Nevertheless, both methods indicated rs12913832 to explain the most variation of the skin color phenotype. Next in order, in RF, there were rs12896399 and rs4911414 for skin color, rs731236, rs12896399 and rs4911414 (above 10% each) for tanning and rs12203592 (14.3%) followed by rs1015362, rs12896399, rs4911414 and rs731236 (above 9% each) for freckling (Fig. 3). For 2-category estimations, BLR prediction rates with the corresponding AUC values for tanning and freckling phenotypes were, respectively, 69.4% (AUC = 0.673) and 52.8% (AUC = 0.537). The low rate of skin color prediction success can be partially explained by small sample size for individuals with dark skin (*n* = 3) in test predictions. Nevertheless, the significantly predictive variants were rs12913832 for all three pigmentation traits, accompanied with rs16891982 for skin color and tanning, rs1015362 and rs4911414 for tanning and rs4911414 and rs12203592 for freckling (Table 4).

*HERC2* together with *OCA2* are found to be the most involved in human pigmentation, especially in the iris and hair color (Walsh et al. 2012; Donnelly et al. 2012). In particular, an intronic variant rs12913832 in *HERC2* acts as a functional enhancer for *OCA2* promoter, therefore, facilitating melanin production (Visser et al. 2012). This variant not only explains the most blue and brown iris color variation but also isconsidered to be associated with skin color showing a strong geographical pattern in genotype distribution across Europe (Bouakaze et al. 2009; Walsh et al. 2012; Duffy et al. 2007; Pośpiech et al. 2014; Lao et al. 2007).

In our study, rs12913832 turned out to explain the most of the variance of skin color and tanning in 2- as well as in 3- and 4-category estimation models. The major lighter color-associated rs12913832-C variant was observed significantly more often in individuals with pale skin than in those with moderate or dark but also in individuals with moderate skin color when compared to those with dark skin shade. The OR values for C and CC variants were even higher for individuals that tanned poorly in comparison with moderate and quick tanning ones (between 3 and 16.7). 90% of subjects with severe sunburns and 66.7% of those with initial sunburns had rs12913832-CC. The association was milder for the freckling phenotype; however, the results were still significant. Our results are similar to those of other authors who demonstrated rs12913832 as one of the strongest and directly associated with melanin production in skin (Walsh et al. 2013; Bouakaze et al. 2009; Lao et al. 2007; Valenzuela et al. 2010; Pneuman et al. 2012; Liu et al. 2015).

The second strongest eye color predictor rs1800407 in *OCA2* is a missense variant that exerts an epistatic effect on rs12913832 (Pośpiech et al. 2014; Frudakis et al. 2003; Crawford et al. 2017). Interestingly, that SNP was one of the least important variants in most models in our study and there were no significant differences in allele or genotype frequencies between distinct phenotypes. One possible reason could be the fact that the minor A allele was only present as the AG heterozygote in the training group in small number of individuals (10%) and the major GG homozygote was a dominant genotype for all phenotypes in this study.

Three SNPs showed strong association with selected skin pigmentation predictions and these were: rs6058017 and rs4911414 in *ASIP* and rs12203592 in *IRF4*. Several SNPs in *ASIP* have been reported to influence skin sensitivity to sun and freckling, namely rs6058017 and two others comprising the ASIP 2-SNP haplotype tagged by rs1015362-C and rs4911414-T (Pośpiech et al. 2014; Eriksson et al. 2010; Sulem et al. 2008). Other authors reported the rs1015362 major C variant to be associated with red hair, severe freckling and high susceptibility to sunburns (Sulem et al. 2008); however, in our training group, the CC homozygote was equally distributed among distinct pigmentation phenotypes and the results were insignificant. On the other hand, rs4911414 seemed to be more involved in skin pigmentation than rs1015362 alone. There were no rs1015362-CC + rs4911414-TT genotypes in the training group and the LD value for the two variants was insignificant, though noticeable (LD = 65; Fig. 2). Rs4911414 explained most of the tanning phenotypes in BLR and the overall importance of rs4911414 alone was around 8% for NN and between 9% and 10.6% for RF. Indeed, there was a noticeable tendency for rs4911414-TT to be more frequent in individuals with lighter skin shades, poorer tanning and freckling. According to other authors, rs4911414-T indeed seemed to be associated with sunburns, freckling and red hair, even to a greater extent than rs6058017 alone (Pośpiech et al. 2014; Eriksson et al. 2010; Sulem et al. 2008). In our study, the importance of rs6058017 and rs4911414 was comparable in the models. However, rs6058017 gave much higher OR results showing strong significant association with the tanning phenotypes, which makes it presumable risk variant only for susceptibility to sunburns prediction. Analogically, rs12203592 in *IRF4* showed a great association exclusively with freckling. It was the first most important variant in RF, NN and BLR. We observed over 20-fold higher prevalence of the minor T allele in severely freckled subjects than in those without freckles, and almost fivefold higher for binomial comparison in favor of general freckling. In this study, there was only one rs12203592-TT subject who was severely freckled, had pale skin shade, initial sunburns and light blue eyes. Although the presence of such an outlier might be affecting the robustness of the model, it may still enrich the model as a representative of genetically and phenotypically rare individual observed in the analyzed population. Likewise, Eriksson et al. observed a strong association between the rs12203592-T variant and freckling trait in northern Europeans (Eriksson et al. 2010), while Duffy et al. (2010) showed that rs12203592-T carriers of Australia were prone to develop a great number of nevi. The same authors stated that the minor T allele was most common in European individuals in comparison with those of African and East Asian descent. The association of rs12203592 with lighter skin and iris color and red hair has also been shown by other authors; however, the freckling trait was not considered as a separate feature (Walsh et al. 2012, 2017; Han et al. 2008). Therefore, rs12203592 might be one of the strongest predictors of the freckling feature, exclusively, in the Polish population.

A three-SNP haplotype (rs7495174-T, rs4778241-G, rs4778138-T) within the intron 1 of *OCA2* has been previously found to be in linkage with blue eye color, lighter hair and skin tones, skin sensitivity to sun exposure and freckling (Duffy et al. 2007; Caliebe et al. 2016; Sulem et al. 2007; Frudakis et al. 2007). In our study, rs7495174 turned out to have a heterozygous status in all individuals. Therefore, we examined the two remaining SNPs and the pattern of association with lighter pigmentation phenotypes was comparable with that of other authors. We observed significantly high OR values especially for skin color and tanning estimations (Supplementary Table 3), although the LD between the two markers was insignificant (LD = 58; Fig. 2) and they explained the average of the pigmentation traits in RF and NN. Interestingly, both rs4778241-T and rs4778138-C variants were more frequent in females than in males in our study; however, females showed higher count of lighter pigmentation phenotypes (Table 1). Although Pulker et al. (2007) demonstrated that females were generally paler than males, it has not been confirmed by any other author. According to Shriver et al. (2003), pigmentation in adults is a stable trait that is independent of environmental factors. However, in our opinion, it is worth taking into account that females are highly influenced by the hormonal factors that can affect the final pigmentation phenotype throughout life.

Five SNPs seemed ambiguous in pigmentation prediction and these were: rs12896399 in *SLC24A*, rs731236 in *VDR*, rs1805007 in *MC1R*, rs16891982 in *SLC45A2*, rs1393350 in *TYR*. In the literature the minor rs12896399-T variant was associated with blue eyes, paler skin and poor tanning ability (Han et al. 2008). Interestingly, in our study, rs12896399 turned out to be the second strongest predictor in RF (Fig. 3), although none of the results of allele and genotype frequencies were significant and the OR values were close to the neutral 1 value for all pigmentation phenotypes. It was alike for rs731236, which was one of the strongest variants, especially for tanning prediction, explaining 11% of the trait in RF. The rs731236-G variant was described to be associated with pale skin and red/light hair by other authors (Pośpiech et al. 2014; Orlow et al. 2017). Although, in our study we observed analogous tendency and the OR values were much higher than for rs12896399, none of the results were statistically significant. On the other hand, rs731236 has been shown to be associated with other variants, such as the *R* variants of *MC1R* in predicting light vs. dark skin and red vs. non-red hair in UK individuals (Walsh et al. 2017), but also influenced sensitivity to sun and freckling in people of Icelandic, North American and Siberian origin (Bouakaze et al. 2009; Valenzuela et al. 2010; Caliebe et al. 2016; Sulem et al. 2007; Myles et al. 2007). *MC1R* is considered as one of the strongest factors in melanin synthesis pathway in Europeans and has been particularly associated with red hair and pale skin, mostly through its interactions with other pigmentation markers including *HERC2*, *OCA2* and *ASIP* (Duffy et al. 2007; Pośpiech et al. 2014; Valenzuela et al. 2010; Caliebe et al. 2016; Lalueza-Fox et al. 2007; Branicki et al. 2009). Interestingly, no strong LD was observed between rs1805007 and other markers in this study and there was only one lighter pigmentation-associated TT variant carrier, who indeed had pale and severe freckled skin, high susceptibility to sunburns, red hair and hazel eyes. Alike in the case of single rs12203592-TT carrier, rs1805007-TT individual was retained in the model. Another ambiguous variant was rs16891982, which explained almost the least in multinomial predictions but was significant in BLR predictions. We observed significantly high OR values for skin color and tanning estimations and the discrepancy between the methods might be the result of the lack of the minor CC carriers and quite small percentage of GC heterozygotes (6.7%) in the training group. Still, even in rs16891982-GC variants, the C allele shifted the balance towards the darker skin shades and better tanning, which was in agreement with other authors (Bouakaze et al. 2009; Stokowski et al. 2007; Valenzuela et al. 2010; Pneuman et al. 2012). For the last SNP of the IrisPlex, rs1393350, we observed a tendency for association with the minor T allele with lighter pigmentation phenotypes, which was in agreement with Sulem et al. (2007). However, despite over 8% contribution to multinomial models and high OR values, none of the results were significant.

Apart from an individual association between a SNP and a phenotype, our modeling results are difficult to compare with that of other authors for several reasons. Firstly, when referring to estimation among major populations on a global level, many authors obtained high prediction values that explained 70–97% of skin pigmentation variation (Lao et al. 2007; Liu et al. 2015; Walsh et al. 2017; Maroñas et al. 2014). Walsh et al. (2017) studied 36 SNPs on 31 world populations and were not only able to distinguish the light and dark skin shades between continental groups but also to separate the subtle variation of skin tones even in 5-category scale. However, when referring to global skin color prediction, the authors used an adequate scaling of skin tones from white to black; while for our Polish population, the grading of the trait was adjusted to the generally white skin color in this geographical region. Therefore, the term “dark” in our study would not be synonymous with the term “dark” in studies considering global skin color. Because of more subtle differences in skin shades within one population, it is easier to assess greater diversity among populations than within one. Myles et al. stated that the skin color is an adaptive trait and there are considerable genetic differences reaching up to 85% between populations and only up to 15% inside a population (Myles et al. 2007). Taking that into account, as it is quite evident to spot significant differences between two or more extreme phenotypic traits, our prediction results assessed for homogeneous population seem quite satisfying. The lowest predictive value in our study was 47.2% for tanning prediction in 4-category scale. In comparison, Valenzuela et al. who examined homogeneous North American population, reached 45.7% of skin color estimation (Valenzuela et al. 2010). Moreover, Maroñas et al. (2014) investigated skin color trait in population of South Asians and Europeans and, surprisingly, several variants previously reported to be associated with pigmentation in various populations turned out not to be significant, including the strongest pigmentation predictor in a worldwide population rs12913832 in *HERC2*.

Secondly, Walsh et al. (2017) considered the skin color as the main pigmentation trait indicating the fact that the actual phenotype might change upon exposure to sun. Our study, on the other hand, proved that distinct SNPs might be associated with tanning capabilities but not with skin color and vice versa, and that the two traits might not even be associated with each other that much (Table 2). The final aim of police investigations is to find an unknown person using EVCs. Still, given the prediction on one’s skin color, it might be inconclusive not knowing what their skin sensitivity to sun exposure is and how much, if at all, the skin shade changes upon tanning. Our study provides additional valuable information on the presumable final phenotype that might be relevant in forensic and anthropologic genetics applications. Importantly, when referring to genotype–phenotype predictions, one should always consider geographical origin of a subject based on mitochondrial DNA testing, which is highly recommended by other authors (Kayser and de Kniff 2011; Pneuman et al. 2012). Lastly, the comparison between our prediction models and that of other authors is limited due to the algorithms used. We tested three different approaches, of which GLM turned out to be the most uncertain for 3- and more-category estimations, while it was a method of choice in prediction modeling used by many authors (Walsh et al. 2013, 2017; Dario et al. 2015; Valenzuela et al. 2010; Han et al. 2008; Maroñas et al. 2014; Liu et al. 2010). Considering categorical data, such as SNPs, logistic regression approach seems not to be the most appropriate mathematical algorithm. Our conclusions were replicated by Pośpiech et al. who evaluated predictive capacity of SNPs using GLM, Neural Network and Classification and Regression Trees algorithms and indicated that GLM was indeed not the best in predictive success (Pośpiech et al. 2015). In addition, none of the authors indicated the hyperparameters used in analyses and on what basis the parameters in their studies were chosen. Therefore, our study elucidates the need for more appropriate analyses for different types of data to increase the forensic investigation efficiency.

Worth mentioning, we spotted differences in allele distribution for six SNPs (rs4778241, rs4778138, rs12896399, rs12203592, rs731236, rs1805007) between our study and the CEU of 1000Genomes study which were all in favor of presumable darker skin pigmentation traits among our study participants than in individuals of general European consent. We also observed different genotype distribution between males and females for two *OCA2* variants in this study which, all together, implies that other genetic variants might be responsible for pigmentation traits in the Polish population. It certainly requires further examination on greater number of individuals, which is a definite drawback of this study. Nevertheless, we assessed to confirm that rs12913832 in the enhancer of *OCA2* seemed to be the strongest variant for skin color, tanning and freckling traits, suggesting it is a strong general pigmentation marker in the Polish population. The other two variants in *ASIP*, rs6058017 and rs4911414, but not rs1015362, were strongly associated exclusively with skin sensitivity to sun exposure, while rs12203592 in *IRF4* turned out to be the strongest freckling predictor. Lastly, the rs4778241 and rs4778138 *OCA2* haplotype and rs16891982 in *SLC45A2* seemed promising for skin color and tanning capabilities in Polish population.

## Electronic supplementary material

Below is the link to the electronic supplementary material.

SNP markers used in this study for skin pigmentation traits predictions. Characteristics for each SNP, PCR and sbe primers with concentration are included 1 (XLSX 15 kb)

The frequency of alleles and genotypes in 13 differentiated SNPs genotyped in this study with the respective training sets of 150 individuals, using the Fisher’s exact test. ^a^ wild homozygote/allele as first; ^b^ number of individuals with a genotype/allele (genotype/allele frequency) 2 (XLSX 16 kb)

The frequency of alleles and genotypes in 13 differentiated SNPs genotyped in this study for 150 training individuals calculated for distinct phenotypes using the Fisher’s exact test. ¹ wild homozygote/allele as first 3 (XLSX 39 kb)

Hyperparameters tested in grid search analysis for GLM, RF and NN algorithms prior to developing the prediction models 4 (XLSX 11 kb)
